# Intermediate-Temperature Creep Deformation and Microstructural Evolution of an Equiatomic FCC-Structured CoCrFeNiMn High-Entropy Alloy

**DOI:** 10.3390/e20120960

**Published:** 2018-12-12

**Authors:** Chengming Cao, Jianxin Fu, Tongwei Tong, Yuxiao Hao, Ping Gu, Hai Hao, Liangming Peng

**Affiliations:** 1 CAS Key Laboratory of Mechanical Behavior and Design of Materials, Department of Modern Mechanics, School of Engineering Science, University of Science and Technology of China, Hefei 230027, China; 2 School of Materials Science and Engineering, Dalian University of Technology, Dalian 116024, China

**Keywords:** high entropy alloy, tensile creep behavior, microstructural evolution, creep mechanism

## Abstract

The tensile creep behavior of an equiatomic CoCrFeNiMn high-entropy alloy was systematically investigated over an intermediate temperature range (500–600 °C) and applied stress (140–400 MPa). The alloy exhibited a stress-dependent transition from a low-stress region (LSR-region I) to a high-stress region (HSR-region II). The LSR was characterized by a stress exponent of 5 to 6 and an average activation energy of 268 kJ mol^−1^, whereas the HSR showed much higher corresponding values of 8.9–14 and 380 kJ mol^−1^. Microstructural examinations on the deformed samples revealed remarkable dynamic recrystallization at higher stress levels. Dislocation jogging and tangling configurations were frequently observed in LSR and HSR at 550 and 600 °C, respectively. Moreover, dynamic precipitates identified as M_23_C_6_ or a Cr-rich σ phase were formed along grain boundaries in HSR. The diffusion-compensated strain rate versus modulus-compensated stress data analysis implied that the creep deformation in both stress regions was dominated by stress-assisted dislocation climb controlled by lattice diffusion. Nevertheless, the abnormally high stress exponents in HSR were ascribed to the coordinative contributions of dynamic recrystallization and dynamic precipitation. Simultaneously, the barriers imposed by these precipitates and severe initial deformation were referred to so as to increase the activation energy for creep deformation.

## 1. Introduction

High-entropy alloys (HEAs), as a novel type of material generally consisting of five or more principle elements, have attracted extensive attention in recent years because of their attractive crystallographic and mechanical properties [1,2,3,4,5,6,7]. Although it is anticipated that simple solid solution phases are easier to form rather than the intermetallic compounds or complex-ordered phases due to their high configurational entropy [8,9], only a few HEAs have exactly single and stable solid solution structures, among which CoCrFeNiMn alloy is widely concerned [10,11,12,13,14]. It possesses a single face-center-cubic (fcc) solid solution phase even after a series of thermomechanical processing [15,16] and its constituent elements have more sluggish diffusion compared to other conventional alloys [17]. These characterizations contribute to excellent mechanical properties with a satisfactory combination between tensile strength and ductility [18,19]. Apart from previous investigations mainly focusing on its temperature dependence of strength [20,21], which provided a fundamental understanding of the strengthening mechanism in fcc solid solution HEAs, recent reports have elucidated the thermally activated process, where dislocation lines overcome nanoscale clusters or short-range orders for controlling the deformation rate [22,23]. This intrinsic mechanism indicated effective heat-resistance during high-temperature plastic deformation processes. Owing to the aforementioned sluggish elemental diffusion, it is optimistic that the CoCrFeNiMn alloy will exhibit a promising perspective in high-temperature applications. 

When referring to high-temperature performance for most alloy systems, creep resistance is one of the most important standards for service life and safety reliability of engineering structures. In particular, it is necessary to evaluate the stress exponents and activation energies to demonstrate the mechanism operative in the power-law creep deformation, as these two parameters are usually employed to predict the steady-state creep strain. However, only quite limited reports on tensile creep properties of HEAs at elevated temperatures have been available. The investigation on creep behavior of AlxCoCrFeNi (x = 0.15, 0.60) alloys using the stress relaxation method [24] demonstrated a lower creep resistance for higher Al content alloy, which was attributed to the easier dislocation cross-slip due to its higher stacking fault energy. The high-stress exponent of 8.8 and activation energy of 334 kJ mol^−1^ were simply explained by the increasing grain boundary in the initial body-center-cubic (bcc) phase. The dislocation configurations and microstructural evolution were not examined to confirm the validity of the proposed creep mechanism. Recently, He et al. [25,26] investigated the high-temperature plastic flow behavior of single-phase FeCoNiMn and precipitation-hardened (FeCoNiCr)_94_Ti_2_Al_4_ HEAs using strain-rate-jump/stress increment tests at different temperatures (750–900 °C). In general, the deformation in these two alloys was divided into two regimes, dependent on the applied strain rate or testing temperatures. Specifically, the obtained higher stress exponent (6–9) and activation energy (>600 kJ mol^−1^) in the latter alloy were proposed to be associated with the strong interactions between dislocations and coherent precipitates, resulting in a threshold stress term in the flow constitutive equation [26]. It should be pointed out that the testing temperatures were up to 750–900 °C and far beyond the softening temperature (~600 °C) of the investigated alloys [19]. From a scientific view, the strain-rate-jump or stress increment test method is inadequate using a single specimen at respective temperatures, as the microstructure during elevated-temperature plastic flow is generally not invariant. Moreover, such plastic-flow behavior is, to some extent, obviously different from creep deformation under a constant applied stress. Consequently, it is still necessary to systematically investigate the intermediate temperature (not higher than 600 °C) creep deformation and examine the microstructural evolution for exploring the intrinsic deformation mechanism of HEAs.

In the present study, the tensile creep tests under constant stress were performed on multiple fine-grained CoCrFeNiMn HEA specimens in the temperature range of 500–600 °C. Specifically, some tests were interrupted at the steady-state stages and cooled quickly to reserve the crept microstructures for examining the microstructural evolution. The objective was to reveal the inherent relationship between microstructural evolution and creep properties of single-phase CoCrFeNiMn high-entropy alloy.

## 2. Experimental Procedures

Ingots with a nominal composition of Co_20_Cr_20_Fe_20_Ni_20_Mn_20_ (in atomic percentage) were prepared by vacuum induction melting the constituent elements with at least 99.9 mass % purity under argon atmosphere. To ensure chemical homogeneity, the ingots were re-melted at least five times and then drop-cast into a rectangular steel mold with a dimension of 120 × 65 × 10 mm^3^. The ingots were homogenized at 1100 °C for 24 h in a vacuum, followed by furnace cooling. Cold rolling was subsequently conducted on these ingots with a reduction of 40% in thickness, with several cross-rolling steps to ensure flatness. The rolled sheets were eventually annealed at 900 °C for 1 h.

Flat dog-boned specimens with a dimension of 25 mm in gauge length and 5.6 mm × 1.5 mm in cross-section were electric-discharge machined for creep tests. The specimens were mechanically ground to 2000-grid sand paper to remove surface asperities. Tensile creep tests were conducted at temperatures of 500, 550, and 600 °C under applied stresses of 140–400 MPa on a CSS-3905 multi-functional testing machine. The temperature was measured with a temperature accuracy of ±1 °C by three thermocouples closely attached to the upper, middle, and lower sections of the specimen, respectively. The creep strain was continuously measured using a Linear Variable Differential Transducer (LVDT) extensometer with a strain resolution of ± 0.1 µm. The acquisition of strain-time data was accomplished by a computer, and data processing was conducted through a computer program. At least seven applied stress levels were chosen to obtain a wide range of creep rates for each testing temperature. Several specimens were subjected to interrupted tests at a steady-state stage and cooled quickly under the load using liquid nitrogen to freeze the structures produced during creep deformation. 

The microstructural observation was conducted using optical microscopy (OM) (AxioImager.A1m, Jena, Germany) after etching in an aqueous solution of HCl+H_2_O_2_+Cu(NO_3_)_2_. The phase constitution was identified by X-ray diffraction (XRD) with Cu-Ka radiation (PANalytical X’pert PRO, Almelo, Netherlands). Thin foils from the interrupted specimens for transmission electron microscope (TEM) observations were twin-jet electropolished in an ethanol solution containing 5% perchloric acid at −25 °C and an applied voltage of 35 V. TEM investigations were conducted on a JEOL JEM2100F microscope operated at 200 kV. Fracture surfaces were examined by a scanning electron microscope (SEM) (XL30 ESEM, Philip, Netherlands) equipped with an energy-dispersive spectrometer (EDS)).

## 3. Results

### 3.1. Initial Microstructures

**Figure 1:**
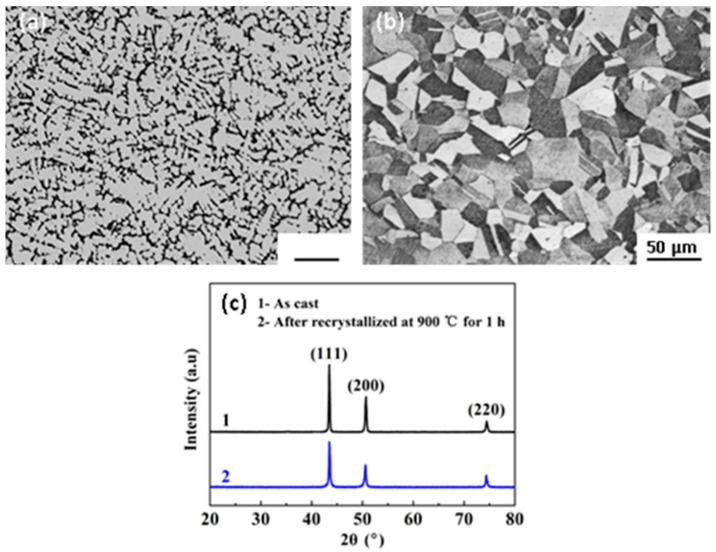
Microstructures of the alloy in different states. (**a**) As-cast, (**b**) thermo-mechanical treatment (cold-rolled and annealed at 900 °C for 1 h), and (**c**) X-ray diffraction (XRD) patterns.

Figure 1 shows the initial microstructures of the alloy in as-cast and annealed states prior to high-temperature creep deformation. It is evident that the cast alloy exhibits typical dendritic and interdendritic structures, caused by the segregation during the quick freezing process in the mold. After being cold-rolled and recrystallized, equiaxed and homogenous grain structures are obtained with many annealing twins visible inside the grains. The average grain size is evaluated to be approximately 25 μm. Energy-dispersive spectroscopy (EDS) analyses demonstrate that different grains have almost identical elemental compositions as Cr = 19.9, Co = 19.9, Fe = 19.9, Ni = 19.8, and Mn=19.5 with a little loss of Mn due to its slight evaporation at an elevated temperature. As depicted by XRD patterns in Figure 1c, the alloy consists of a single fcc solid solution phase without precipitates or intermetallic compounds in both cast and recrystallized states. Furthermore, almost no peak shift is observed in the two states, with a cell parameter of a = 0.361 nm for the fcc phase. The present results are different from those observed in the Al_0.3_CoCrFeNi HEA where precipitation of nanometer scale-ordered L12, B2, and sigma phases occurred in the case of different thermo-mechanical processing routes [27].

### 3.2. Steady-State Creep Deformation Behavior

**Figure 2:**
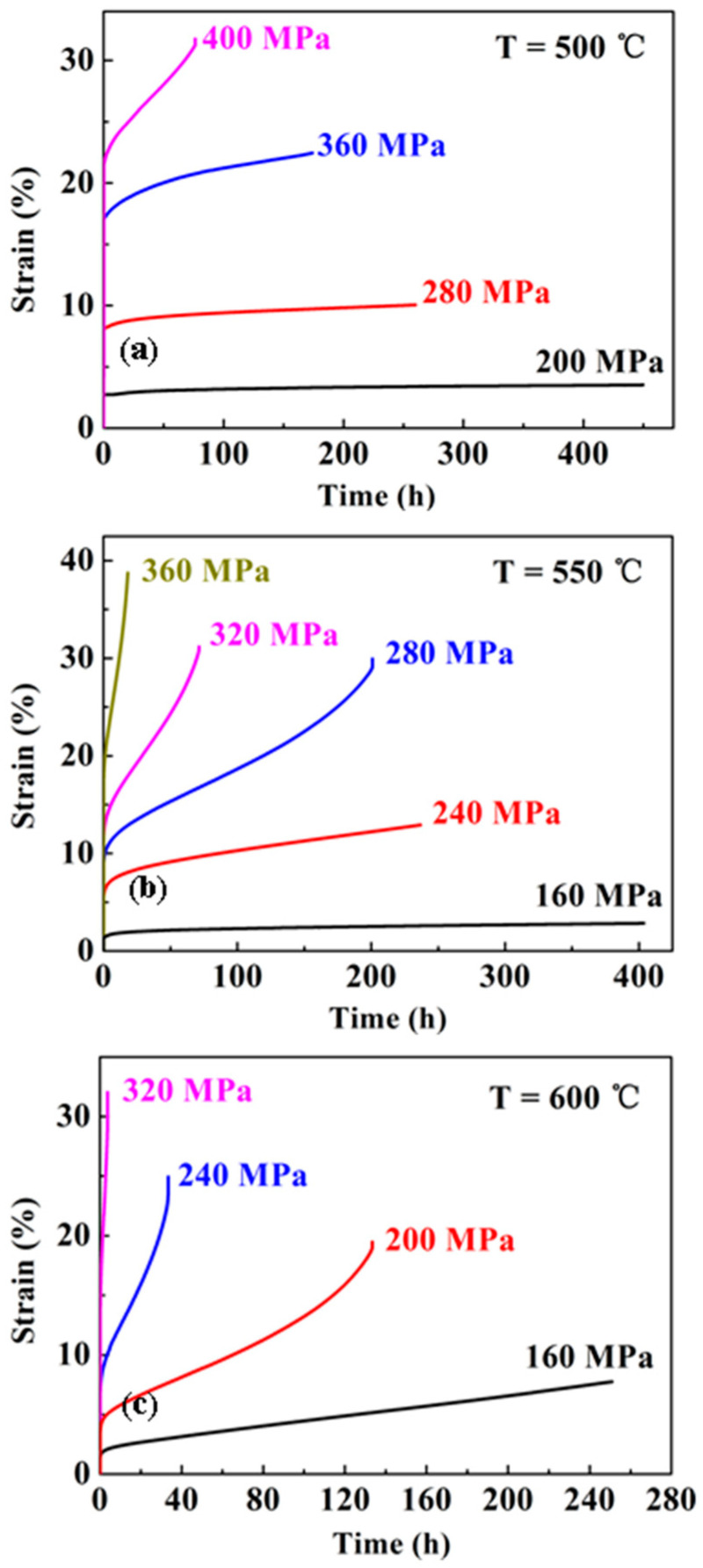
Selected creep strain versus time at (**a**) 500 °C, (**b**) 550 °C, and (**c**) 600 °C.

Figure 2 shows the selected creep curves of the alloy at 500–600 °C under different stress levels. It can be found that each of the individual curves exhibits a rather short primary creep stage and a relatively long steady-state region where the creep strain increases linearly with time, especially under lower stress levels. Necking and fracture of the specimens do occur, and hence, a tertiary stage of the curves is recorded in cases of high stress, where the creep rate accelerates with time until the final fracture occurs.

**Figure 3:**
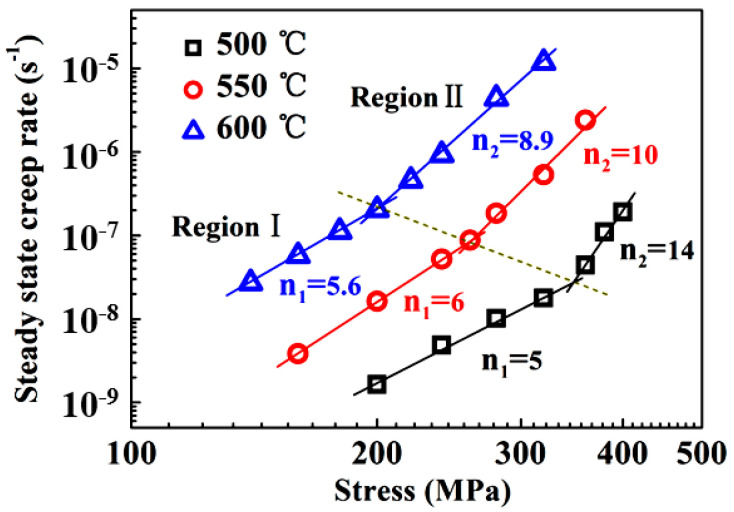
Dependence of creep rates on applied stress showing the transition in stress exponents.

**Figure 4:**
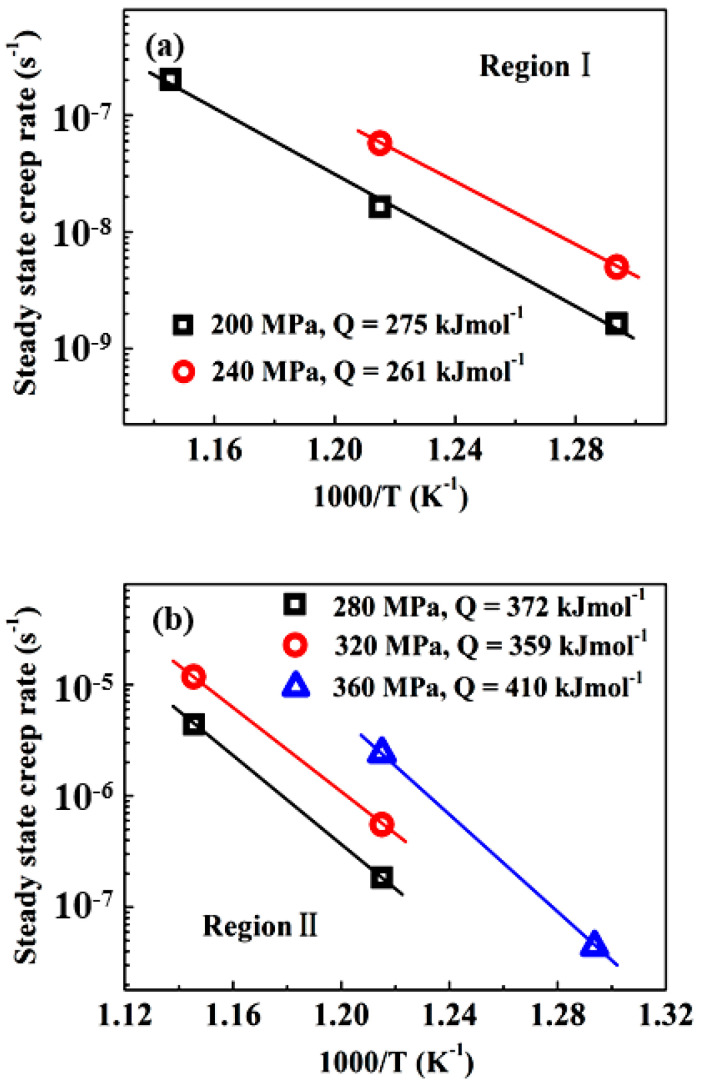
Arrhenius plot of steady-state creep rate versus temperature to determine the activation energy for (**a**) low-stress region I and (**b**) high-stress region II.

It is generally accepted that the steady-state creep rate $\dot{\varepsilon }$ of metallic materials can be correlated with the applied stress σ using the well-known power-law equation as follows [28]:

(1) $$\dot{\varepsilon }=\frac{AD_{0}Gb}{kT}{\left(\frac{b}{d}\right)}^{p}{\left(\frac{\sigma }{G}\right)}^{n}\exp(-\frac{Q}{RT})$$

where A is a material-dependent constant, G the shear modulus, b the Burgers vector, $D_{0}$ the frequency factor, k the Boltzmann’s constant, T the absolute temperature, d the grain size of polycrystalline materials, Q the activation energy for creep deformation, and p and n the grain size and stress exponents, respectively. The stress exponent n and activation energy value Q are determined according to Equation (1) by plotting the steady-state creep rate against applied stress on a double logarithmic scale and the reciprocal of the absolute temperatures 1/T at constant stress levels on a semi-logarithmic scale, as shown in Figure 3 and Figure 4, respectively. It is evident that the data exhibit two distinct regions. In the low-stress region (subsequently denoted as LSR-region I), the stress exponent values vary in the range of 5 to 6, and the average activation energy Q takes a value of 268 kJ mol^−1^. On the contrary, in the high-stress region (subsequently denoted as HSR-region II), the stress exponents are in the range of 8.9–14, and the activation energy is Q = 359–410 kJ/mol with an average value of 380 kJ mol^−1^. The applied stress at which the transition occurred increased from 200 to 350 MPa, with the temperature decreasing from 600 to 500 °C.

**Figure 5:**
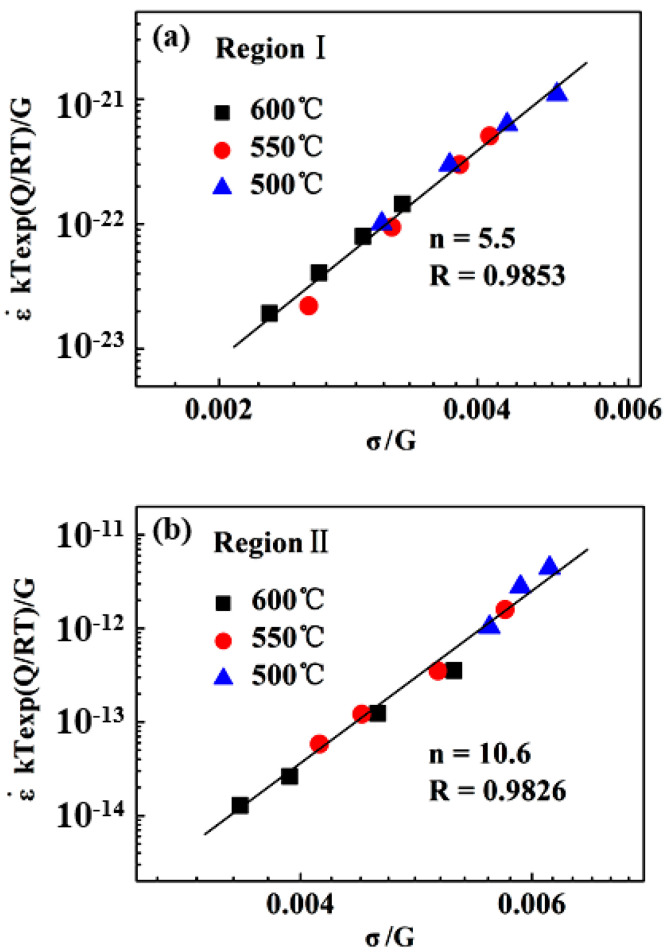
Normalized creep rate versus shear modulus-compensated stress for (**a**) low-stress region I and (**b**) high-stress region II.

It should be noted that the average activation energy of 268 kJ mol^−1^ in LSR-region I is only slightly lower than the range of activation energies for lattice diffusion of the constituent elements in the alloy (288–317 kJ mol^−1^ [17]), and thus can be considered to be comparable to the lattice diffusion of the constituent elements in the alloy. In addition, this value is also quite close to those (284–333 kJ mol^−1^) reported for the steady-state flow behavior of this alloy at higher temperatures and lower stress levels [26]. The two characteristic parameters suggest a dislocation-climb mechanism operative in LSR. Nevertheless, the abnormally high stress exponent in HSR-region II is obviously beyond the values of 3 to 5 responsible for the dislocation glide or climb mechanism [29,30] reported in some Mg [31], Al [32], and Ti [33,34] alloys. The underlying operative mechanism for HSR creep deformation in the present alloy will be addressed in the following section. In order to eliminate the influence of temperature on creep rate, the normalized creep rates $\dot{\varepsilon }kT\exp(Q/RT)/G$ versus the shear modulus-compensated stresses σ/G were plotted in Figure 5, where activation energies Q were taken to be 268 kJ mol^−1^ and 380 kJ mol^−1^ for region I and region II, respectively, and the shear modulus $G=85-16/(e^{448/T}-1)$ [35]. It is evident that almost all data points in the two stress regions at different temperatures can be represented by a single line with a respective slope of 5.5 and 10.6 for LSR and HSR. This strongly implies that creep deformation in both regions may be controlled by lattice diffusion of constituent elements in the alloy. However, it should be noted that the correlation between $\dot{\varepsilon }kT\exp(Q/RT)/G$ and σ/G in HSR still yields an abnormally high stress exponent. 

### 3.3. Crept Microstructures

**Figure 6:**
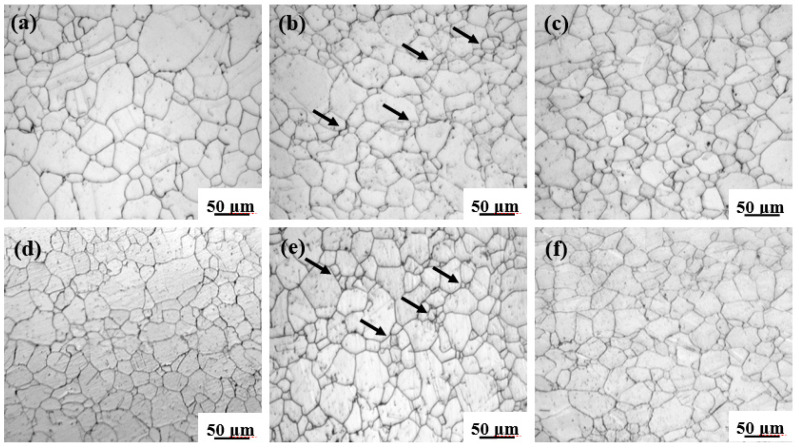
Optical microstructures showing the evolution of grain morphology after being crept at (**a**) 500 °C/200MPa, (**b**) 500 °C/320MPa, (**c**) 500 °C/400MPa, (**d**) 600 °C/140MPa, (**e**) 600 °C/200MPa, and (**f**) 600 °C/320MPa.

Figure 6 shows the microstructures of the interrupted samples at different temperatures and stress levels to illustrate the evolution of grain morphologies. Compared to the initial microstructure of the alloy, obvious coarsening can be observed when the specimens are subjected to low stress levels (500 °C/200MPa and 600 °C/140MPa), as depicted in Figure 6a,d. Similar grain coarsening phenomena are also visible at intermediate stress levels (500 °C/320MPa and 600 °C/200MPa) near the transition stress indicated in Figure 3. However, the volume fraction of small equiaxed grains increases at the coarse grain boundaries (indicated by arrows in Figure 6b,e), revealing strong evidence of the occurrence of dynamic recovery and recrystallization during creep deformation. Moreover, higher stress levels (500 °C/400MPa and 600 °C/320MPa) lead to the formation of completely recrystallized and refined microstructures (Figure 6c,f). The resulting average grain sizes are statistically evaluated to be approximately 56, 45, and 22 μm at 500 °C, whereas 48, 43, and 31 μm at 600 °C under different chosen stress levels.

**Figure 7:**
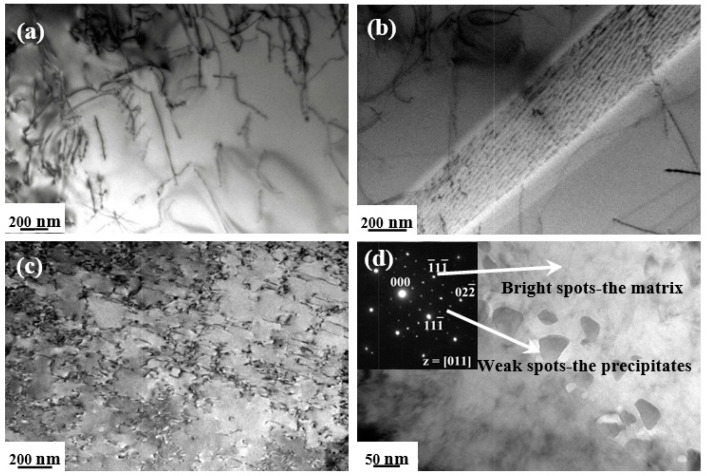
Transmission electron microscope (TEM) images of dislocation substructures in the interrupted specimens after being crept at 500 °C under (**a**) and (**b**) 200 MPa (low-stress region (LSR)); (**c**) and (**d**) 400 MPa (high-stress region (HSR)).

**Figure 8:**
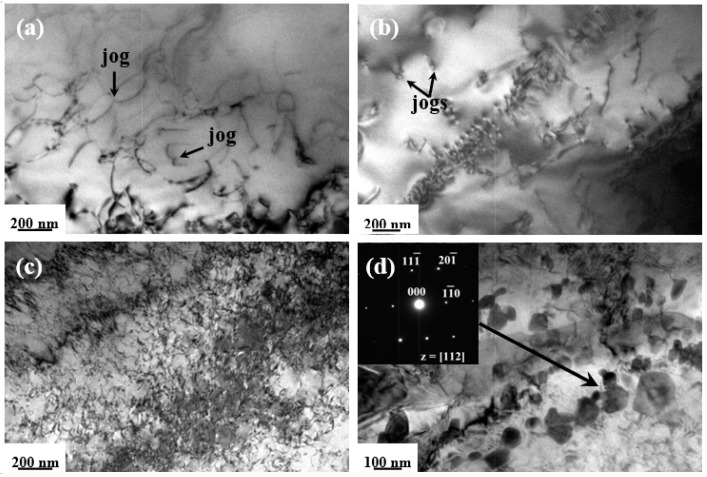
TEM images of dislocation substructures in the interrupted specimens after being crept at 550 °C under (**a**) and (**b**) 160 MPa (LSR); (**c**) and (**d**) 360 MPa (HSR).

**Figure 9:**
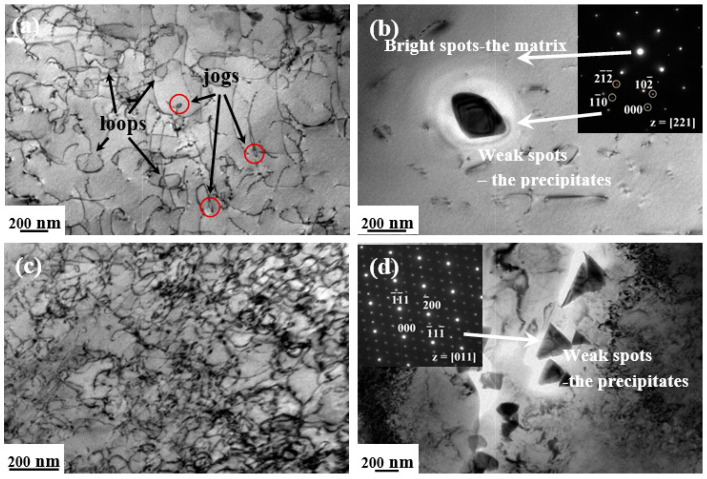
TEM images of dislocation substructures in the interrupted specimens after being crept at 600 °C under (**a**) and (**b**) 140 MPa (LSR); (**c**) and (**d**) 320 MPa (HSR).

The dislocation substructures are shown in Figure 7, Figure 8 and Figure 9, with selected area electron diffraction (SAED) patterns inserted to identify the precipitations. In general, the dislocation densities in HSR were distinctly higher than those in LSR at three testing temperatures. After being deformed at 500 °C/200 MPa (LSR), only a few short straight dislocation segments were displayed inside the grains (Figure 7a), and in particular, several lath-shaped areas were formed with closely-spaced dislocations arranged (Figure 7b). However, the substructures reveal increasing dislocation activity in LSR with increasing temperature, as the dislocations are extensively curved or even looped (Figure 8a and Figure 9a). Apart from the limited number of alignments of pile-ups in Figure 8b, it is noted that cusped configurations are frequently observed inside grains with the bowed segments on either side. Such cusped configurations may be attributed to intrinsic pinning due to the occurrence of jogs along the screw dislocations [34]. In contrast, the dislocations in HSR exhibit a higher density of configurations. Numerous short straight segments are tangled and tend to form cell substructures at 500 °C/400 MPa (Figure 7c). However, the dislocations at 550 °C/360 MPa show a relatively homogeneous distribution still with high densities of curved dislocations tangling with each other (Figure 8c). The tangled dislocations at 600 °C/320 MPa are severely curved, with a large number of loops formed in a wide range of sizes (Figure 9c).

Another striking aspect of microstructural evolution relies on the precipitation of dispersoids during a long-term deformation process at elevated temperatures. On the whole, considerable precipitates of irregular shape were predominantly observed at the grain boundaries in HSR. The sizes of these precipitates were within a range of 50–200 nm and increased with testing temperature. In cases of 500 °C/400MPa and 600 °C/320MPa (Figure 7d and Figure 9d), quantitative micro-EDS analysis indicates that the chemical compositions (at. %) of the precipitates take almost identical values of Cr = 26.6, Mn = 20.2, Fe = 18.9, Co = 17.5, Ni =16.6 (in at. %) and a minor amount of carbon. The SAED pattern along the [011] zone axis exhibits intense diffraction spots from the fcc matrix, accompanied by weak spots from the precipitates. The crystal structure of these precipitates is identified as fcc M_23_C_6_ carbide with a lattice parameter of 1.06 nm, which is consistent with both the general identifications of grain-boundary precipitates in austenitic steel [36] and the second phase observed in coarse-grained CoCrFeNiMn alloy after being subjected to prolonged exposures at 700 °C [37]. The tiny presence of carbon in the alloy may have originated from either the potential contamination of starting materials, or the melting system. Nevertheless, as depicted in Figure 8d and Figure 9b for 550 °C/360MPa and 600 °C/140MPa, the micro-EDS analysis demonstrates chemical compositions (at %) of the precipitates with Cr = 48.8, Mn = 15.5, Fe = 14.0, Co = 11.5, and Ni = 10.2. The present results are quite consistent with previous reports for the prolonged annealing CrMnFeCoNi system [38,39], where the precipitates were identified as a quinary variant of the binary Cr-Fe σ phase. The interplanar spacings are calculated to be d_1_/d_2_/d_3_ = 0.648/0.402/0.337 nm and show a satisfactory agreement with the values of d_1_/d_2_/d_3_ = 0.648/0.383/0.330 nm for (1$\bar{1}$0)/(11$\bar{1}$)/(20$\bar{1}$) lattice planes in the [112] zone axes for the σ-FeCrMo phase [40]. Accordingly, the precipitates in the 50 °C/360MPa crept alloy are concluded to be Cr-rich tetragonal σ phase with lattice parameters of a/c = 0.916/0.509 nm. Similarly, the weak spots along the [221] zone axes in the SAED pattern in Figure 9b are also indicated to be very close to the Cr-rich σ phase, with lattice parameters of a/c = 0.895/0.473 nm.

### 3.4. Fractographs

**Figure 10:**
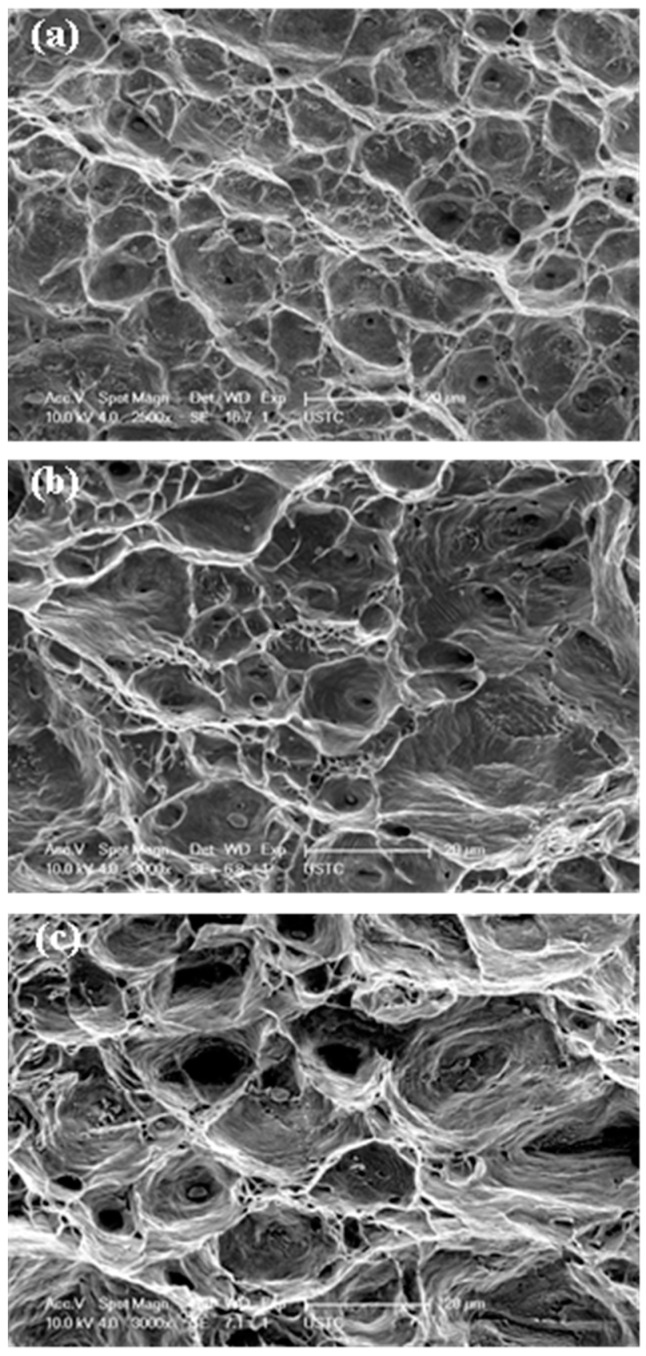
Microstructural features of the fracture surfaces after being deformed at high stress levels. (**a**) 500 °C/400MPa, (**b**) 550 °C/360MPa, and (**c**) 600 °C/320MPa.

All the fractographs in Figure 10 for HSR from 500 °C to 600 °C exhibit typical ductile rupture, where both the width and depth of dimples increase with temperature. However, slip striations around the dimples become notable at 600 °C. Meanwhile, a small number of particles identified as Mn-containing oxides by EDS are visible on the fracture surfaces, which is attributed to an in-situ oxidation during fracture process [26]. It is worth noting that the crept samples fracture in a ductile transgranular manner at high stress, which is contrary to common knowledge, as large amounts of brittle are generally considered to be destructive to the stability of grain boundaries during the tertiary creep stage [41,42]. The ductile creep failure may be associated with the dislocation interactions during high-temperature deformation, which contribute to the progressive generation of vacancies and nucleation of voids.

## 4. Discussion

The observed jog configurations are indicative of a dislocation climb process, and thus the stress-assisted dislocation climb controlled by lattice diffusion may be the operative creep mechanism of the present high-entropy alloy in LSR. Contrary to LSR, creep deformation in HSR exhibits abnormally high stress exponents of 8.9–14 and an average activation energy of 380 kJ mol^−1^. Combined with the dislocation configurations in Figure 8c and Figure 9c, the same creep mechanism may also be considered rate-controlling. The unusually high stress exponents and activation energy in several traditional alloys have been frequently described using the power-law breakdown equation [43,44,45,46]. However, no deviation from the linear relationship on double-logarithmic curves of $\log\dot{\varepsilon }$~logσ were observed, and therefore, the power-law breakdown assumption may be inappropriate for the present alloy.

It is documented that dynamic recrystallization during the creep process could cause an increase in the stress exponent [47,48,49]. Recrystallization is generally accompanied by intense diffusion of solutes, resulting in a refined-grain microstructure and a larger opportunity for grain boundary sliding. Under such circumstances, the creep rate would increase with decreasing grain size [50]. As shown in Figure 6, obvious dynamic recrystallization occurred in the present CoCrFeNiMn alloy in HSR. In fact, low stacking-fault energy of alloys is conducive to promoting recrystallization [51]. Based on Equation (1) where p is generally equal to 2 for a dislocation-controlled creep, an increase in the stress exponent Δn due to dynamic recrystallization is expressed as [47]:

(2) $$\Delta n=n_{app}-n=\frac{2\ln(d_{1}/d_{2})}{\ln(\sigma_{2}/\sigma_{1})}$$

where $n_{app}$ is the experimentally measured apparent stress exponent, n is the stress exponent prior to the occurrence of recrystallization, $d_{1}$ and $d_{2}$ denote the average grains size before and after dynamic recrystallization, and $\sigma_{1}$ and $\sigma_{2}$ are the corresponding stress levels. Using the relevant evaluated grain sizes, the Δn values were calculated to be 6.5 and 1.4 for 500 °C and 600 °C, respectively. The corresponding modified stress exponents n then fall down to ~7.5. However, this value is still higher than those for traditional solid solution alloys, which implies that the sole recrystallization is insufficient and additional mechanisms should be involved to be responsible for the entire increase in stress exponents.

TEM observations provide clear evidence of dynamic precipitation in HSR. As a result, precipitation hardening may have contributed to the high stress exponents [25]. In general, the precipitates are predominantly formed along the grain boundaries and would exert a boundary obstacle stress $\sigma_{bo}$, which prevent the annihilation of moving dislocations at the boundaries. The stress controlling the dislocation velocity is reduced to an effective value obtained by subtracting the stress necessary for overcoming precipitates from the applied stress. Equation (1) is then replaced by [52,53]:

(3) $$\dot{\varepsilon }=\frac{AD_{0}Gb}{kT}{\left(\frac{b}{d}\right)}^{p}{\left(\frac{\sigma -\sigma_{b0}}{G}\right)}^{n}\exp(-\frac{Q}{RT})$$

By re-plotting the double-logarithm relationship between $\dot{\varepsilon }/\exp(-Q/RT)$ and $(\sigma -\sigma_{bo})/G$ in HSR, the stress exponents can then be decreased by subtracting the precipitation-hardening portion. Unfortunately, it is difficult to precisely estimate the values of boundary obstacle stress and the decrease in the stress exponent, due to the ambiguous interaction between dynamic precipitation and recrystallization. Nevertheless, combinative effects of precipitation hardening and recrystallization can provide reasonable insights into the intrinsic reason for the abnormal stress exponents and activation energy for creep in CoCrFeNiMn alloy. Moreover, the estimated abnormal activation energy in HSR might also stem partially from precipitation hardening [22]. The nano-sized dispersoids could effectively fasten the grain boundaries, and thus extra activation energy is needed to overcome the barriers for recrystallization and further growth of grains. In addition, the samples experience drastic plastic deformation in a short time to establish a constant stress state for subsequent creep deformation. As a result, some grains are severely elongated, which, in turn, exerts an extra influence on increasing the barriers for dynamic recovery and recrystallization.

## 5. Conclusions

The intermediate-temperature creep behavior and microstructural evolution of CoCrFeNiMn HEA have been studied. Optical micrographs and TEM images of the dislocation substructure after creep deformation revealed the effects of dynamic recrystallization and dynamic precipitation on the deformation mechanisms. The following conclusions can be drawn:

1. An obvious transition in stress exponent and average activation energy was observed: At low stresses, the values were approximately 5 to 6 and 268 kJ/mol, whereas at high-stress regions, the corresponding values were 8.9–14 and 380 kJ/mol. Stress-assisted dislocation climb controlled by lattice diffusion is suggested as a possible rate-dominating mechanism at low stresses.
2. At high stresses, obvious dynamic recrystallization occurred, leading to the refinement of average grain size. Simultaneously, nano-sized M_23_C_6_ carbides and the Cr-rich tetragonal σ phase were dynamically precipitated. High density of tangled and curved dislocation substructures was observed.
3. The anomalously high stress exponent and activation energy in HSR were attributed to the combinative effects of dynamic recrystallization and precipitation. In particular, the precipitates act as barriers for dislocation motion via producing boundary obstacle stress, resulting in high activation energy for creep deformation. However, lattice-diffusion controlled dislocation climb is still responsible for the deformation mechanism in HSR.
